# Discretization of Learned NETT Regularization for Solving Inverse Problems

**DOI:** 10.3390/jimaging7110239

**Published:** 2021-11-15

**Authors:** Stephan Antholzer, Markus Haltmeier

**Affiliations:** Department of Mathematics, University of Innsbruck, Technikerstrasse 13, 6020 Innsbruck, Austria; stephan.antholzer@uibk.ac.at

**Keywords:** deep learning, inverse problems, discretization of NETT, regularization, convergence analysis, learned regularizer, limited data, photoacoustic tomography

## Abstract

Deep learning based reconstruction methods deliver outstanding results for solving inverse problems and are therefore becoming increasingly important. A recently invented class of learning-based reconstruction methods is the so-called NETT (for Network Tikhonov Regularization), which contains a trained neural network as regularizer in generalized Tikhonov regularization. The existing analysis of NETT considers fixed operators and fixed regularizers and analyzes the convergence as the noise level in the data approaches zero. In this paper, we extend the frameworks and analysis considerably to reflect various practical aspects and take into account discretization of the data space, the solution space, the forward operator and the neural network defining the regularizer. We show the asymptotic convergence of the discretized NETT approach for decreasing noise levels and discretization errors. Additionally, we derive convergence rates and present numerical results for a limited data problem in photoacoustic tomography.

## 1. Introduction

In this paper, we are interested in neural network based solutions to inverse problems of the form

(1) $$\;\mathrm{Find}\;x\;\mathrm{from}\;\mathrm{data}\;\;y^{\delta }=Ax+\eta \;.$$

Here A is a potentially non-linear operator between Banach spaces X and Y, $y^{\delta }$ are the given noisy data, *x* is the unknown to be recovered, η is the unknown noise perturbation and δ≥0 indicates the noise level. Numerous image reconstruction problems, parameter identification tasks and geophysical applications can be stated as such inverse problems [1,2,3,4]. Special challenges in solving inverse problems are the non-uniqueness of the solutions and the instability of the solutions with respect to the given data. To overcome these issues, regularization methods are needed, which select specific solutions and at the same time stabilize the inversion process.

### 1.1. Reconstruction with Learned Regularizers

One of the most established class of methods for solving inverse problems is variational regularization where regularized solutions are defined as minimizers of the generelaized Tikhonov functional [2,5,6]

(2) $$T_{y^{\delta },\alpha }:X\to \left[0,\infty \right]:x\mapsto D\left(Ax,y^{\delta }\right)+\alpha R\left(x\right)\;.$$

Here D is a distance like function measuring closeness of the data, R a regularization term enforcing regularity of the minimizer and α is the regularization parameter. Taking minimizers of this functional as regularized solution is also called (generalized) Tikhonov regularization. In the case that D and the regularizer are defined by the Hilbert space norms, (2) is classical Tikhonov regularization for which the theory is quite complete [1,7]. In particular, in this case, convergence rates, which name quantitative estimates for the distance between the true noise-free solution and regularized solutions from noisy data, are well known. Convergence rates for non-convex regularizers are derived in [8].

Typical regularization techniques are based on simple hand crafted regularization terms such as the total variation $∥f{∥}_{\mathrm{TV}}=\int \left|\nabla f\right|$ or quadratic Sobolev norms ${∥\nabla f∥}_{2}^{2}=\int {\left|\nabla f\right|}^{2}$ on some function space. However, these regularizers are quite simplistic and might not well reflect the actual complexity of the underlying class of functions. Therefore, recently, it has been proposed and analyzed in [9] to use machine learning to construct regularizers in a data driven manner. In particular, the strategy in [9] is to construct a data-driven regularizer via the following consecutive steps:

(T1) Choose a family of desired reconstructions ${\left(x_{i}\right)}_{i=1}^{n}$.
(T2) For some B:Y→X, construct undesired reconstructions ${\left(BAx_{i}\right)}_{i=1}^{n}$.
(T3) Choose a class ${\left(\Phi_{\theta }\right)}_{\theta \in \Theta }$ of functions (networks) $\Phi_{\theta }:X\to X$.
(T4) Determine $\theta^{⋆}\in \Theta$ with $\Phi_{\theta^{⋆}}\left(x_{i}\right)≃x_{i}\wedge \Phi_{\theta^{⋆}}\left(BAx_{i}\right)≃x_{i}$.
(T5) Define R(x)=r(x,Φ(x)) with $\Phi =\Phi_{\theta^{⋆}}$ for some r:Y×Y→[0,∞].

For imaging applications, the function class ${\left(\Phi_{\theta }\right)}_{\theta \in \Theta }$ can be chosen as convolutional neural networks which have demonstrated to give powerful classes of mappings between image spaces. The function *r* measures distance between a potential reconstruction *x* and the output of the network Φ(x), and possibly contains additional regularization [10,11]. According to the training strategy in item (T4) the value of the regularizer will be small if the reconstruction is similar to elements in ${\left(x_{i}\right)}_{i=1}^{n}$ and large for elements in ${\left(BAx_{i}\right)}_{i=1}^{n}$. A simple example that we will use for our numerical results is the learned regularizer $R\left(x\right)=∥{x-\Phi (x)∥}^{2}+{∥x∥}_{\mathrm{TV}}$.

Convergence analysis and convergence rates for NETT (which stands for Network Tikhonov; referring to variants of (2), where the regularization term is given by a neural network) as well as training strategies have been established in [9,11,12]. A different training strategy for learning a regularizer has been proposed in [13,14]. Note that learning the regularizer first and then minimizing the Tikhonov functional is different from variational and iterative networks [15,16,17,18,19,20] where an iterative scheme is applied to enroll the functional $D_{\theta }\left(Ax,y^{\delta }\right)+\alpha R_{\theta }\left(x\right)$ which is then trained in an end to end fashion. Training the regularizer first has the advantage of being more modular, sharing some similarity with plug and play techniques [21], and the network training is independent of the forward operator A. Moreover, it enables to derive a convergence analysis as the noise level tends to zero and therefore comes with theoretical recovery guarantees.

### 1.2. Discrete NETT

The existing analysis of NETT considers minimizers of the Tikhonov functional (2) with regularizer of the form R(x)=r(x,Φ(x)) before discretization, typically in an infinite dimensional setting. However, in practice, only finite dimensional approximations of the unknown, the operator and the neural network are given. To address these issues, in this paper, we study discrete NETT regularization which considers minimizers of

(3) $$T_{y^{\delta },\alpha ,n}:X_{n}\to Y:x\mapsto D\left(A_{n}x,y^{\delta }\right)+\alpha R_{n}\left(x\right)\;.$$

Here ${\left(X_{n}\right)}_{n\in N}$, ${\left(A_{n}\right)}_{n\in N}$ and ${\left(R_{n}\right)}_{n\in N}$ are families of subspaces of $X_{n}\subseteq X$, mappings $A_{n}:X\to Y$ and regularizers $R_{n}:X\to \left[0,\infty \right]$, respectively, which reflect discretization of all involved operations. We present a full convergence analysis as the noise level δ converges to zero and n,α are chosen accordingly. Discretization of variational regularization has studied in [22] for the case that D is given by the norm distance and the regularizer R is taken convex and fixed. However, in the case of discrete NETT regularization it is natural to consider the case where the regularization depends on the discretization as regularization is learned in a discretized setting based on actual data. For that purpose our analysis includes non-convex regularizers that are allowed to depend on the discretization and the noise level.

### 1.3. Outline

The convergence analysis including convergence rates is presented in Section 2. In Section 3 we will present numerical results for a non-standard limited data problem in photoacoustic tomography that can be considered as simultaneous inpainting and artifact removal problem. We conclude the paper with a short summary and conclusion presented in Section 4.

## 2. Convergence Analysis

In this section we study the convergence of (3) and derive convergence rates.

### 2.1. Well-Posedness

First we state the assumptions that we will use for well-posedness (existence and stability) of minimizing NETT.

**Assumption** **1** (Conditions for well-posedness).

*(W1)*  *X, Y are Banach spaces, X reflexive, D⊆X weakly sequentially closed.*
*(W2)*  *The distance measure D:Y×Y→[0,∞] satisfies*
  *(a)*  *$\exists \tau \geq 1:\forall y_{1},y_{2},y_{3}\in Y:D\left(y_{1},y_{2}\right)\leq \tau D\left(y_{1},y_{3}\right)+\tau D\left(y_{3},y_{2}\right)$.*
  *(b)*  *$\forall y_{1},y_{2}\in Y:D\left(y_{1},y_{2}\right)=0\Leftrightarrow y_{1}=y_{2}$.*
  *(c)*  *$\forall y,\tilde{y}\in Y:D\left(y,\tilde{y}\right)<\infty \wedge ∥\tilde{y}-y_{k}∥\to 0\Rightarrow D\left(y,y_{k}\right)\to D\left(y,\tilde{y}\right)$.*
  *(d)*  *$\forall y\in Y:∥y_{k}-y∥\to 0\Rightarrow D\left(y_{k},y\right)\to 0$.*
  *(e)*  *D is weakly sequentially lower semi-continuous (wslsc).*
*(W3)*  *R:X→[0,∞] is proper and wslsc.*
*(W4)*  *A:D⊆X→Y is weakly sequentially continuous.*
*(W5)*  *$\forall y,\alpha ,C:\{x\in X|T_{y,\alpha }\leq C\}$ is nonempty and bounded.*
*(W6)*  *${\left(X_{n}\right)}_{n\in N}$ is a sequence of subspaces of X.*
*(W7)*  *${\left(A_{n}\right)}_{n\in N}$ is a family of weakly sequentially continuous $A_{n}:D\to Y$.*
*(W8)*  *${\left(R_{n}\right)}_{n\in N}$ is a family of proper wslsc regularizers $R_{n}:X\to \left[0,\infty \right]$.*
*(W9)*  *$\forall y,\alpha ,C,n:\{x\in X_{n}|T_{y,\alpha ,n}\leq C\}$ is nonempty and bounded.*

Conditions (W2)–(W5) are quite standard for Tikhonov regularization in Banach spaces to guarantee the existence and stability of minimizers of the Tikhonov functional and the given conditions are similar to [2,8,9,10,12,23,24]. In particular, (W2) describes the properties that the distance measure D should have. Clearly, the norm distance on Y fulfills these properties. Moreover, (W2a) holds for the norm with τ=1 since it then corresponds to the triangle inequality. Item (W2c) is the continuity of D(y,·) while (W2d) considers the continuity of D(·,y) at *y*. While (W2c) is not needed for existence and convergence of NETT it is required for the stability result as shown in [10] (Example 2.7). On the other hand (W2e) implies that the Tikhonov functional is wslsc which is needed for existence. Assumption (W5) is a coercivity condition; see [9] (Remark 2.4f.) on how to achieve this for a regularizer defined by neural networks. Item (W8) poses some restrictions on the regularizers. For NETT this is not an issue as neural networks used in practice are continuous. Note that for convergence and convergence rates we will require additional conditions that concern the discretization of the reconstruction space, the forward operator and regularizer.

The references [8,9,10,23] all consider general distance measures and allow non-convex regularizers. However, existence and stability of minimizing (2) are shown under assumptions slightly different from (W1)–(W5). Below we therefore give a short proof of the existence and stability results.

**Theorem** **1** (Existence and Stability). *Let Assumption 1 hold. Then for all $y^{\delta }\in Y$, α>0, n∈N the following assertions hold true:*

*(a)*  *$\mathrm{argmin}T_{y,\alpha ,n}\neq \emptyset$.*
*(b)*  *Let ${\left(y_{k}\right)}_{k\in N}\in Y^{N}$ with $y_{k}\to y$ and consider $x_{k}\in \mathrm{argmin}T_{y_{k},\alpha ,n}$.*
  - *${\left(x_{k}\right)}_{k\in N}$ has at least one weak accumulation point.*
  - *Every weak accumulation point ${\left(x_{k}\right)}_{k\in N}$ is a minimizer of $T_{y,\alpha ,n}$.*
*(c)*  *The statements in (a),(b) also hold for $T_{y,\alpha }$ in place of $T_{y,\alpha ,n}$,*

**Proof.**  Since (W1), (W6)–(W9) for $T_{y,\alpha ,n}$ when n∈N are fixed give the same assumption as (W1), (W3)–(W5) for the non-discrete counterpart $T_{y,\alpha }$, it is sufficient to verify (a), (b) for the latter. Existence of minimizers follows from (W1), (W2e), (W3)–(W5), because these items imply that the $T_{y,\alpha }$ is a wslsc coercive functional defined on a nonempty weakly sequentially closed subset of a reflexive Banach space. To show stability one notes that according to (W2a) for all x∈X we have

$$\begin{array}{c} D\left(Ax_{k},y\right)+\alpha R\left(x_{k}\right)\leq \tau \left(D\left(Ax_{k},y_{k}\right)+\alpha R\left(x_{k}\right)\right)+\tau D\left(y,y_{k}\right)\\ \;\;\;\;\;\;\;\;\;\;\;\;\;\;\;\;\;\;\leq \tau \left(D\left(Ax,y_{k}\right)+\alpha R\left(x\right)\right)+\tau D\left(y,y_{k}\right)\;. \end{array}$$

According to (W2c), (W2d), (W5) there exists x∈X such that the right hand side is bounded, which by (W5) shows that ${\left(x_{k}\right)}_{k}$ has a weak accumulation point. Following the standard proof [2] (Theorem 3.23) shows the weak accumulation points ${\left(x_{k}\right)}_{k}$ are minimizers of $T_{y,\alpha }$. This uses the fact that the weak topology is indeed weaker than the norm topology, and that the involved functionals are wslsc.    □

In the following we write $x_{\alpha ,n}^{\delta }$ for minimizers of $T_{y^{\delta },\alpha ,n}$. For y∈Y we call $x^{+}\in \mathrm{argmin}\left\{R\left(x\right)|x\in X\wedge Ax=y\right\}$ an R-minimizing solution of Ax=y.

**Lemma** **1** (Existence of R-minimizing solutions). *Let Assumption 1 hold. For any y∈A(D) an R-minimizing solution of Ax=y exists. Likewise, if n∈N and $y\in A_{n}\left(D\right)$ an $R_{n}$-minimizing solution of $A_{n}x=y$ exists.*

**Proof.**  Again is is sufficient the verify the claim for R-minimizing solution. Because y∈A(D), the set $A^{-1}\left(\left\{y\right\}\right)=\left\{x\in X|Ax=y\right\}$ is non-empty. Hence we can choose a sequence ${\left(x_{k}\right)}_{k\in N}$ in $A^{-1}\left(\left\{y\right\}\right)$ with $R\left(x_{k}\right)\to \inf\left\{R\left(x\right)|x\in X\wedge Ax=y\right\}$. Due to (W2b), ${\left(x_{k}\right)}_{k\in N}$ is contained in {x∈X∣D(A(x),y)+αR(x)≤C} for some C>0 which is bounded according to (W5). By (W1) X is reflexive and therefore ${\left(x_{k}\right)}_{k\in N}$ has a weak accumulation point $x^{+}$. From (W1), (W4), (W3) we conclude that $x^{+}$ is an R-minimizing solution of Ax=y. The case of $R_{n}$-minimizing solutions follows analogous.    □

### 2.2. Convergence

Next we proof that discrete NETT converges as the noise level goes to zero and the discretization as well as the regularization parameter are chosen properly. We write $D_{n,M}:=\left\{x\in D\cap X_{n}|R_{n}\left(x\right)\leq M\right\}$ and formulate the following approximation conditions for obtaining convergence.

**Assumption** **2** (Conditions for convergence).

*Element $x^{+}\in D$ satisfies the following for all M>0:*

*(C1)*  *$\exists \left(z_{n}\right)\in \prod_{n\in N}\left(D\cap X_{n}\right)$ with $\lambda_{n}:=\left|R_{n}\left(z_{n}\right)-R\left(x^{+}\right)\right|\to 0$.*
*(C2)*  *$\rho_{n}:=\sup_{x\in D_{n,M}}\left|R_{n}\left(x\right)-R\left(x\right)\right|\to 0$.*
*(C3)*  *$\gamma_{n}:=D\left(A_{n}z_{n},Ax^{+}\right)\to 0$.*
*(C4)*  *$a_{n}:=\sup_{x\in D_{n,M}}\left|D\left(A_{n}x,Ax^{+}\right)-D\left(Ax,Ax^{+}\right)\right|\to 0$.*

Conditions (C1) and (C3) concerns the approximation of the true unknown *x* with elements in the discretization space, that is compatible with the discretization of the forward operator and regularizer. Conditions (C2) and (C4) are uniform approximation properties of the operator and the regularizer on $R_{n}$-bounded sets.

**Theorem** **2** (Convergence). *Let (W1)–(W9) hold, y∈A(D) and let $x^{+}$ be an R-minimizing solution of Ax=y that satisfies (C1)–(C4). Moreover, suppose ${\left(\delta_{k}\right)}_{k\in N}\in {\left(0,\infty \right)}^{N}$ converges to zero and ${\left(y_{k}\right)}_{k\in N}\in Y^{N}$ satisfies $D\left(y,y_{k}\right)\leq \delta_{k}$. Choose ${\left(\alpha_{k}\right)}_{k\in N}$ and ${\left(n_{k}\right)}_{k\in N}$ such that as k→∞ we have*

(4) $$\begin{array}{cc} \; & \alpha_{k}\to 0 \end{array}$$

(5) $$\begin{array}{cc} \; & n_{k}\to \infty \end{array}$$

(6) $$\begin{array}{cc} \; & \left(\delta_{k}+D\left(A_{n_{k}}z_{n_{k}},y\right)\right)/\alpha_{k}\to 0\;. \end{array}$$

*Then for $x_{k}\in \mathrm{argmin}T_{y_{k},\delta_{k},n_{k}}$ the following hold:*

*(a)*  *${\left(x_{k}\right)}_{k\in N}$ has a weakly convergent subsequence ${\left(x_{\sigma (k)}\right)}_{k\in N}$*
*(b)*  *The weak limit of ${\left(x_{\sigma (k)}\right)}_{k\in N}$ is an R-minimizing solution of Ax=y.*
*(c)*  *$R_{\sigma (k)}\left(x_{\sigma (k)}\right)\to R\left(x^{⋆}\right)$, where $x^{⋆}$ is the weak limit of ${\left(x_{\sigma (k)}\right)}_{k\in N}$.*
*(d)*  *If the R-minimizing solution of Ax=y is unique, then ${\left(x_{k}\right)}_{k\in N}⇀x^{+}$.*

**Proof.**  For convenience and some abuse of notation we use the abbreviations $R_{k}:=R_{n_{k}}$, $A_{k}:=A_{n_{k}}$, $a_{k}:=a_{n_{k}}$, $z_{k}:=z_{n_{k}}$ and $\rho_{k}:=\rho_{n_{k}}$. Because $x_{k}$ is a minimizer of the discrete NETT functional $T_{y_{k},\delta_{k},n_{k}}$ by (W2) we have

$$\begin{array}{cc} D\left(A_{k}x_{k},y_{k}\right) & +\alpha_{k}R_{k}\left(x_{k}\right)\leq D\left(A_{k}z_{k},y_{k}\right)+\alpha_{k}R_{k}\left(z_{k}\right)\\ & \leq \tau D\left(A_{k}z_{k},y\right)+\tau D\left(y,y_{k}\right)+\alpha_{k}R_{k}\left(z_{k}\right)=\tau D\left(A_{k}z_{k},y\right)+\tau \delta_{k}+\alpha_{k}R_{k}\left(z_{k}\right) \end{array}$$

According to (C1), (4), we get

(7) $$\begin{array}{c} D(A_{k}x_{k},y_{k})\leq \tau (D\left(A_{k}z_{k},y\right)+\delta_{k})\;, \end{array}$$

(8) $$\begin{array}{c} R_{k}\left(x_{k}\right)\leq \tau \;\cdot \;\frac{D\left(A_{k}z_{k},y_{k}\right)+\delta_{k}}{\alpha_{k}}+R_{k}\left(z_{k}\right)\;. \end{array}$$

According to (C1), (C3), (5), (6) the right hand side in (7) converges to zero and the right hand side in (8) to $R(x_{+})$. Together with (C2) we obtain $R(x_{k})\leq R_{k}\left(x_{k}\right)+\rho_{k}\to R\left(x_{+}\right)$ and $D\left(Ax_{k},y\right)\leq D\left(A_{k}x_{k},y\right)+a_{k}\leq \tau D\left(A_{k}x_{k},y\right)+a_{k}+\tau \delta_{k}\to 0$. This shows that ${\left(D\left(Ax_{k},y\right)+R\left(x_{k}\right)\right)}_{k\in N}$ is bounded and by (W1), (W9) there exists a weakly convergent subsequence ${\left(x_{\sigma (k)}\right)}_{k\in N}$. We denote the weak limit by $x^{⋆}\in X$. From (W2), (W4) we obtain Ax=y. The weak lower semi-continuity of R assumed in (W3) shows

$$\begin{array}{c} R\left(x^{⋆}\right)\leq \underset{k}{lim inf}R\left(x_{\sigma (k)}\right)\leq \underset{k}{lim sup}R\left(x_{\sigma (k)}\right)\\ \;\;\;\;\;\;\;\;\;\;\;\;\;\;\;\;\;\leq \underset{k}{lim sup}\left(R_{\sigma (k)}\left(x_{\sigma (k)}\right)+\rho_{k}\right)\leq R\left(x^{+}\right)\;. \end{array}$$

Consequently, $x^{⋆}$ is an R-minimizing solution of Ax=y and $R\left(x_{\sigma (k)}\right)\to R\left(x^{⋆}\right)$. If the R-minimizing solution is unique then $x^{+}$ is the only weak accumulation point of ${\left(x_{k}\right)}_{k\in N}$ which concludes the proof.    □

### 2.3. Convergence Rates

Next we derive quantitative error estimates (convergence rates) in terms of the absolute Bregman distance. Recall that a function R:X→[0,∞] is Gâteaux differentiable at some $x^{⋆}\in X$ if the directional derivative $R^{\prime }\left(x^{⋆}\right)\left(h\right):=\left(R\left(x^{⋆}+th\right)-R\left(x^{⋆}\right)\right)/t$ exist for every h∈X. We denote by $R^{\prime }\left(x^{⋆}\right)$ the Gâteaux derivative of R at *x*. In [9] we introduced the absolute Bregman distance $B_{R}\left(\;\;\cdot \;\;,x^{⋆}\right):X\to \left[0,\infty \right]$ of a Gâteaux differentiable functional R:X→[0,∞] at $x^{⋆}\in X$ with respect to R defined by

(9) $$\forall x\in X:B_{R}\left(x,x^{⋆}\right):=\left|R\left(x\right)-R\left(x^{⋆}\right)-R^{\prime }\left(x^{⋆}\right)\left(x-x^{⋆}\right)\right|\;.$$

We write $\sup_{y^{\delta }}H\left(y^{\delta }\right):=\sup\left\{H\left(y^{\delta }\right)|y^{\delta }\in X\wedge D\left(Ax^{+},y^{\delta }\right)\leq \delta \right\}$. Convergence rates in terms of the Bregman distance are derived under a smoothness assumption on the true solution in the form of a certain variational inequality. More precisely we assume the following:

**Assumption** **3** (Conditions for convergence rates).

*Element $x^{+}\in D$ satisfies the following for all M,δ>0:*

*(R1)*  *Items (C1), (C2) hold.*
*(R2)*  *$\gamma_{n,\delta }:=\sup_{y^{\delta }}\left|D\left(A_{n}z_{n},y^{\delta }\right)-D\left(Ax^{+},y^{\delta }\right)\right|\to 0$.*
*(R3)*  *$a_{n,\delta }:=\sup_{y^{\delta }}\sup_{x\in D_{n,M}}\left|D\left(A_{n}x,y^{\delta }\right)-D\left(Ax,y^{\delta }\right)\right|\to 0$.*
*(R4)*  *R is Gâteaux differentiable at $x^{+}$*
*(R5)*  *There exist a concave, continuous, strictly increasing φ:[0,∞)→[0,∞) with φ(0)=0 and ϵ,β>0 such that for all x∈X*
$$|R\left(x\right)-R\left(x^{+}\right)|\leq \epsilon \Rightarrow \beta B_{R}\left(x,x^{+}\right)\leq R\left(x\right)-R\left(x^{+}\right)+\varphi \left(D(Ax,Ax^{+})\right)\;.$$

According to (R5) the inverse function $\varphi^{-1}:\left[0,\infty \right)\to \left[0,\infty \right)$ exists and is convex. We denote by $\varphi^{-*}\left(s\right):=\sup\left\{s\;t-\varphi^{-1}\left(t\right)|t\geq 0\right\}$ its Fenchel conjugate.

**Proposition** **1** (Error estimates). *Let y∈A(D) and $x^{+}$ be an R-minimizing solution of Ax=y such that (W1)–(W9) and (R1)–(R5) are satisfied. For $y^{\delta }\in Y$ with $D(y,y^{\delta })\leq \delta$ let $x_{\alpha ,n}^{\delta }\in \mathrm{argmin}T_{y^{\delta },\alpha ,n}$. Then for sufficient small δ,α>0 and sufficiently large n∈N, we have the error estimate*

(10) $$B_{R}\left(x_{\alpha ,n}^{\delta },x^{+}\right)\leq \frac{a_{n,\delta }+\gamma_{n,\delta }+\delta }{\alpha }+\rho_{n}+\lambda_{n}+\varphi \left(\tau \delta \right)+\frac{\varphi^{-*}\left(\tau \alpha \right)}{\tau \alpha }\;.$$

**Proof.**  According to Theorem 2 we can assume $|R\left(x_{\alpha ,n}^{\delta }\right)-R\left(x^{+}\right)|<\epsilon$ and with (R5) we obtain

$$\begin{array}{cc} \; & \alpha \beta B_{R}\left(x_{\alpha ,n}^{\delta },x^{+}\right)\\ & \leq \alpha R\left(x_{\alpha ,n}^{\delta }\right)-\alpha R\left(x^{+}\right)+\alpha \varphi \left(D\left(Ax_{\alpha ,n}^{\delta },y\right)\right)\\ & \leq \alpha R_{n}\left(x_{\alpha ,n}^{\delta }\right)-\alpha R_{n}\left(z_{n}\right)+\alpha \rho_{n}+\alpha \lambda_{n}+\alpha \varphi \left(D\left(Ax_{\alpha ,n}^{\delta },y\right)\right)\\ & \leq D\left(A_{n}z_{n},y^{\delta }\right)-D\left(A_{n}x_{\alpha ,n}^{\delta },y^{\delta }\right)+\alpha \rho_{n}+\alpha \lambda_{n}+\alpha \varphi \left(D\left(Ax_{\alpha ,n}^{\delta },y\right)\right)\\ & \leq \delta -D\left(Ax_{\alpha ,n}^{\delta },y^{\delta }\right)+\gamma_{n,\delta }+a_{n,\delta }+\alpha \rho_{n}+\alpha \lambda_{n}+\alpha \varphi \left(\tau \delta \right)+\alpha \varphi \left(\tau D\left(Ax_{\alpha ,n}^{\delta },y^{\delta }\right)\right)\\ & \leq \delta +\gamma_{n,\delta }+a_{n,\delta }+\alpha \rho_{n}+\alpha \lambda_{n}+\alpha \varphi \left(\tau \delta \right)+\tau^{-1}\varphi^{-*}\left(\tau \delta \right)\;. \end{array}$$

For the second inequality we used (C1) and (C2). We have $D\left(A_{n}x_{\alpha ,n}^{\delta },y^{\delta }\right)+\alpha R_{n}\left(x_{\alpha ,n}^{\delta }\right)\leq D\left(A_{n}z_{n},y^{\delta }\right)+\alpha R_{n}\left(z_{n}\right)$ and thus we get an estimate for $R_{n}\left(x_{\alpha ,n}^{\delta }\right)-R_{n}\left(z_{n}\right)$ which we used for the third inequality. For the next inequality we used (R2) and (R3). Finally we used Young’s inequality $\alpha \varphi \left(\tau t\right)\leq t+\tau^{-1}\varphi^{-*}\left(\tau \alpha \right)$ for the last step.    □

**Remark** **1.** *The error estimate (10) includes the approximation quality of the discrete or inexact forward operator $A_{n}$ and the discrete or inexact regularizer $R_{n}$ described by $a_{n,\delta }$ and $\rho_{n}$, respectively. What might be unexpected at first is the inclusion of two new parameters $\lambda_{n}$ and $\gamma_{n,\delta }$. These factors both arise from the approximation of X by the finite dimensional spaces $X_{n}$, where $\gamma_{n,\delta }$ reflects approximation accuracy in the image of the operator A and $\lambda_{n}$ approximation accuracy with respect to the true regularization functional R. Note that in the case where the forward operator, the regularizer, and the solution space X are given precisely, we have $a_{n,\delta }=\gamma_{n,\delta }=\lambda_{n}=\rho_{n}=0$. In this particular case we recover the estimate derived for the NETT in [9].*

**Theorem** **3** (Convergence rates). *Let the assumptions of Proposition 1 hold and consider the parameter choice rule α(δ)≍δ/φ(δ) and let the approximation errors satisfy $a_{n,\delta }+\gamma_{n,\delta }=O\left(\delta \right)$, $\rho_{n}+\lambda_{n}=O\left(\varphi \left(\tau \delta \right)\right)$. Then we have the convergence rate*

(11) $$B_{R}\left(x_{\alpha (\delta ),n(\delta )}^{\delta },x^{+}\right)=O\left(\varphi \left(\tau \delta \right)\right)\;.$$

**Proof.**  Noting that $\varphi^{-*}\left(\tau \delta /\varphi \left(\tau \delta \right)\right)/\delta$ remains bounded as δ→0, this directly follows from Proposition 1.    □

Next we verify that a variational inequality of the form (R5) is satisfied with $\varphi \left(t\right)=c\sqrt{t}$ under a typical source like condition.

**Lemma** **2** (Variational inequality under source condition). *Let R, A be Gâteaux differentiable at $x^{+}\in X$, consider the distance measure $D\left(y_{1},y_{2}\right)={∥y_{1}-y_{2}∥}^{2}$ and assume there exist $\eta \in X^{⋆}$ and $c_{1},c_{2},\epsilon >0$ with $c_{1}∥\eta ∥<1$ such that for all x∈X with $|R\left(x\right)-R\left(x^{+}\right)|\leq \epsilon$ we have*

(12) $$\begin{array}{cc} \; & R^{\prime }\left(x^{+}\right)=A^{\prime }{\left(x^{+}\right)}^{*}\eta \\ \; & ∥Ax-Ax^{+}-A^{\prime }\left(x^{+}\right)\left(x-x^{+}\right)∥\leq c_{1}B_{R}\left(x,x^{+}\right)\\ \; & R\left(x^{+}\right)-R\left(x\right)\leq c_{2}∥Ax-Ax^{+}∥\;. \end{array}$$

*Then (R5) holds with $\varphi \left(t\right)=\left(∥\eta ∥+2c_{2}\right)\sqrt{t}$ and $\beta =1-c_{1}∥\eta ∥$.*

**Proof.**  Let x∈X with $|R\left(x\right)-R\left(x^{+}\right)|\leq \epsilon$. Using the Cauchy-Schwarz inequality and Equation (12), we can estimate

$$\begin{array}{cc} |\langle R^{\prime }\left(x^{+}\right),x-x^{+}\rangle |\leq & ∥A^{\prime }\left(x^{+}\right)\left(x-x^{+}\right)∥∥\eta ∥\\ \leq & ∥Ax-Ax^{+}∥∥\eta ∥+∥Ax-Ax^{+}-A^{\prime }\left(x^{+}\right)\left(x-x^{+}\right)∥∥\eta ∥\\ \leq & ∥Ax-Ax^{+}∥∥\eta ∥+c_{1}∥\eta ∥B_{R}\left(x,x^{+}\right)\;. \end{array}$$

Additionally, if $R\left(x\right)\geq R\left(x^{+}\right)$, we have $|R\left(x\right)-R\left(x^{+}\right)|=R\left(x\right)-R\left(x^{+}\right)$, and on the other hand if $R\left(x\right)<R\left(x^{+}\right)$, we have $|R\left(x\right)-R\left(x^{+}\right)|\leq R\left(x\right)-R\left(x^{+}\right)+2\left(R\left(x^{+}\right)-R\left(x\right)\right)\leq R\left(x\right)-R\left(x^{+}\right)+2c_{2}∥Ax-Ax^{+}∥$. Putting this together we get

$$\begin{array}{cc} B_{R}\left(x,x^{+}\right)\leq |R\left(x\right)- & R\left(x^{+}\right)\left|+\right|\langle R^{\prime }\left(x^{+}\right),x-x^{+}\rangle |\\ & \leq R\left(x\right)-R\left(x^{+}\right)+\left(∥\eta ∥+2c_{2}\right)∥Ax-Ax^{+}∥+c_{1}∥\eta ∥B_{R}\left(x,x^{+}\right)\;, \end{array}$$

and thus $\left(1-c_{1}∥\eta ∥\right)B_{R}\left(x,x^{+}\right)\leq R\left(x\right)-R\left(x^{+}\right)+\left(∥\eta ∥+2c_{2}\right)∥Ax-Ax^{+}∥$.    □

**Corollary** **1** (Convergence rates under source condition). *Let the conditions of Lemma 2 hold and suppose*

$$\begin{array}{cc} \; & \alpha \left(\delta \right)≍\sqrt{\delta }\\ \; & |R_{n(\delta )}\left(z_{n(\delta )}\right)-R\left(x^{+}\right)|=O\left(\sqrt{\delta }\right)\\ \; & \sup\left\{\left|R_{n(\delta )}\left(x\right)-R\left(x\right)\right||x\in D_{n(\delta ),M}\right\}=O\left(\sqrt{\delta }\right)\\ \; & ∥A_{n(\delta )}z_{n(\delta )}-Ax^{+}∥=O\left(\sqrt{\delta }\right)\\ \; & \sup\left\{∥A_{n(\delta )}x-Ax∥|x\in D_{n(\delta ),M}\right\}=O\left(\sqrt{\delta }\right)\\ \; & \sup\{∥A_{n(\delta )}x∥|x\in D_{n(\delta ),M}\}<\infty \;. \end{array}$$

*Then we have the convergence rates result*

(13) $$B_{R}\left(x_{\alpha (\delta ),n(\delta )}^{\delta },x^{+}\right)=O\left(\sqrt{\delta }\right)\;.$$

**Proof.**  Follows from Theorem 3 and Lemma 2. Note that we use ∥·∥ in the theorem, while $D\left(y_{1},y_{1}\right)={∥y_{1}-y_{2}∥}^{2}$ uses the squared norm ${∥\;\;\cdot \;\;∥}^{2}$ and thus the approximation rates for the terms concerning $A_{n(\delta )}$ are order $\sqrt{\delta }$ instead of δ as in Theorem 3.    □

In Corollary 1, the approximation quality of the discrete operator $A_{n}$ and the discrete and inexact regularization functional $R_{n}$ need to be of the same order.

## 3. Application to a Limited Data Problem in PAT

Photoacoustic Tomography (PAT) is an emerging non-invasive coupled-physics biomedical imaging technique with high contrast and high spatial resolution [25,26]. It works by illuminating a semi-transparent sample with short optical pulses which causes heating of the sample followed by expansion and the subsequent emission of an acoustic wave. Sensors on the outside of the sample measure the acoustic wave and these measurements are then used to reconstruct the initial pressure $f:R^{d}\to R$, which provides information about the interior of the object. The cases d=2 and d=3 are relevant for applications in PAT. Here we only consider the case d=2 and assume a circular measurement geometry. The 2D case arises for example when using integrating line detectors in PAT [26].

### 3.1. Discrete Forward Operator

The pressure data $p:R^{2}\times \left[0,\infty \right)\to R$ satisfies the wave equation $\left(\partial_{t}^{2}-\Delta \right)p\left(r,t\right)=0\;\mathrm{for}\;\left(r,t\right)\in R^{2}\times \left(0,\infty \right)$ with initial data p(·,0)= and $\partial_{t}p\left(\;\;\cdot \;\;,0\right)=0$. In the case of circular measurement geometry one assumes that *f* vanishes outside the unit disc $D_{1}:=\{r\in R^{2}|∥r∥<1\}$ and the measurement sensors are located on the boundary $\partial D_{1}=S^{1}$. We assume that the phantom will not generate any data for some region $I\subseteq D_{1}$, for example when the acoustic pressure generated inside *I* is too small to be recorded. This masked PAT problem consists in the recovery of the function *f* from sampled noisy measurements of $g=W(1_{I^{c}}f)$ where W denotes the solution operator of the wave equation and ${𝟙}_{I^{c}}$ the indicator function on $I^{c}:=R^{2}\setminus I$. Note that the resulting inverse problem can be seen of the combination of an inpainting problem and in inverse problems for the wave equation.

In order to implement the PAT forward operator we use a basis ansatz $f\left(r\right)=\sum_{i=1}^{N\times N}x_{i}\psi \left(r-r_{i}\right)$ where $x_{i}\in R$ are basis coefficients and $\psi :R^{2}\to R$ a generalized Kaiser-Bessel (KB) and $r_{i}=\left(i-1\right)/N$ with $i=\left(i_{1},i_{2}\right)\in {\left\{1,\cdots ,N\right\}}^{2}$. The generalized KB functions are popular in tomographic inverse problems [27,28,29,30] and denote radially symmetric functions with support in $D_{R}$ defined by

(14) $$\psi \left(r\right):={\left(1-{∥r∥}^{2}/R^{2}\right)}^{m/2}\;\frac{I_{m}\left(\gamma \sqrt{1-{∥r∥}^{2}/R^{2}}\right)}{I_{m}\left(\gamma \right)}\;\mathrm{for}∥r∥\leq R\;.$$

Here $I_{m}$ is the modified Bessel function of the first kind of order n∈N and the parameters γ>0 and *R* denote the window taper and support radius, respectively. Since W is linear we have $Wf=\sum_{i=1}^{N\times N}x_{i}W\left(\psi \left(\;\;\cdot \;\;-r_{i}\right)\right)$. For convenience we will use a pseudo-3D approach where use the 3D solution of Wψ for which there exists an analytical representation [29]. Denote by $s_{k}$ uniformly spaced sensor locations on $S^{1}$ and by $t_{j}>0$ uniformly sampled measurement times in [0,2]. Define the $N_{t}N_{s}\times N^{2}$ model matrix by $W_{N_{t}\left(k-1\right)+j,N\left(i_{1}-1\right)+i_{2}}=W\left(\psi \left(\;\;\cdot \;\;-r_{i}\right)\right)\left(s_{k},t_{j}\right)$ and an $N^{2}\times N^{2}$ diagonal matrix by ${\left(M_{I}\right)}_{N\left(i_{1}-1\right)+i_{2},N\left(i_{1}-1\right)+i_{2}}=1$ if $r_{i}\in I^{c}$ and zero otherwise. Let $WM_{I}=U\Sigma V^{⊺}$ be the singular valued decomposition. We then consider the discrete forward matrix $A=U\Sigma_{⋆}V^{⊺}$ where $\Sigma_{⋆}$ is the diagonal matrix derived from Σ by setting singular values smaller than some $\sigma_{⋆}$ to zero. This allows us to easily calculate $A^{+}=V\Sigma_{⋆}^{+}U^{⊺}$ where $\Sigma_{⋆}^{+}$ is calculated by inverting all diagonal elements of $\Sigma_{⋆}$ that are greater than zero. In our experiments we use $N=N_{t}=128$, $N_{s}=150$ and take *I* fixed as a diagonal stripe of width 0.34.

### 3.2. Discrete NETT

**Figure 1:**
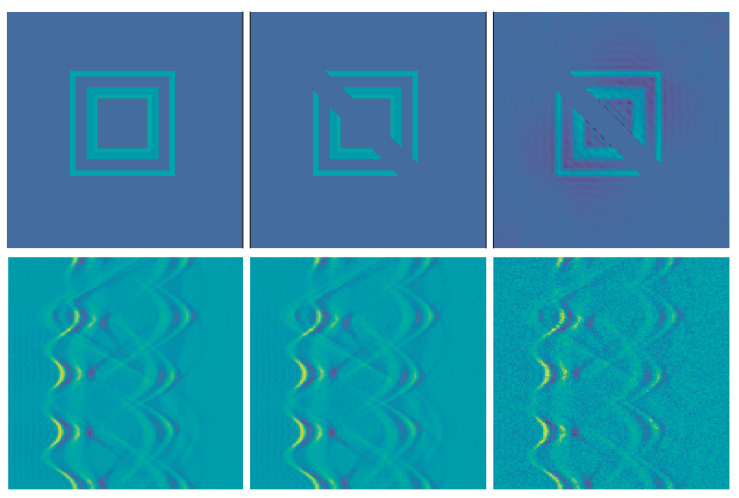
Top from left to right: phantom, masked phantom, and initial reconstruction $A^{+}Ax$. The difference between the phantoms on the left and the middle one shows the mask region $I\subseteq D_{1}$ where no data is generated. Bottom from left to right: data without noise, low noise σ=0.01, and high noise σ=0.1.

We consider the discrete NETT with discrepancy term $D\left(Ax,y^{\delta }\right)={∥Ax-y^{\delta }∥}_{2}^{2}/2$ and regularizer given by

(15) $$R^{\left(m\right)}\left(x\right)=∥{x-\Phi^{\left(m\right)}\left(x\right)∥}_{2}^{2}+\beta {∥\nabla x∥}_{1,\epsilon }\;,$$

where $∥{\nabla x∥}_{1,\epsilon }:=\sum_{i_{1},i_{2}=1}^{128}\sqrt{{\left|r_{i_{1}+1,i_{2}}-r_{i}\right|}^{2}+{\left|r_{i_{1},i_{2}+1}-r_{i_{1},i_{2}}\right|}^{2}+\epsilon^{2}}$ with ϵ>0 is a smooth version of the total variation [31] and $\Phi^{\left(m\right)}$ is a learnable network. We take $\Phi^{\left(m\right)}$ as the U-Net [32] with residual connection, which has first been applied to PAT image reconstruction in [33]. Here *m* stands for the number of down-/upsampling steps performed in the U-Net (the original one had m=4 i.e., m∈N. This means that larger *m* yield a deeper network with more parameters. We generate training data that consist of square shaped rings with random profile and random location. See Figure 1 for an example of one such phantom (note that all plots in signal space use the same colorbar) and the corresponding data. We get a set of phantoms $x_{1},\cdots ,x_{1000}$ and corresponding basic reconstructions $h_{a}:=A^{+}\left(Ax_{a}+\eta_{a}\right)$, where $A^{+}$ is the pseudo-inverse and $\eta_{a}$ is Gaussian noise with standard deviation of $\sigma ∥{Ax_{a}∥}_{\infty }$ with σ=0.01. The networks are trained by minimizing $\sum_{a=1}^{1000}{∥\Phi^{\left(m\right)}\left(h_{a}\right)-x_{a}∥}_{1}+\gamma {∥\Phi^{\left(m\right)}\left(x_{a}\right)-x_{a}∥}_{1}$ where we used the Adam optimizer with learning rate 0.01 and γ=0.1. The considered loss is that we want the trained regularizer to give small values for $x_{a}$ and large values for $h_{a}$. The strategy is similar to [9] but we use the final output of the network for the regularizer as proposed in [34]. To minimize (15) we use Algorithm 1 which implements a forward-backward scheme [35]. The most expensive step of this algorithm is the matrix inversion but since we use constant stepsize one also has to option to only calculate the inverse of the matrix once and reuse it. Thus one only has to perform two matrix-vector multiplications which are of the order $O(N^{2}N_{t}N_{s})$ and $O(N^{4})$ since $N_{2}$ is the dimension of our phantoms. On the other hand calculating the gradient has similar complexity than applying the neural network which is in the order $O(F^{2}LN^{2})$ with *F* the number of convolution channels and *L* the number of layers.
**Algorithm 1:** NETT optimization.
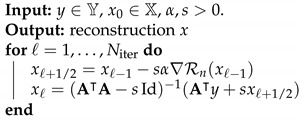


### 3.3. Numerical Results

For the numerical results we train two regularizers $R^{\left(1\right)}$ and $R^{\left(3\right)}$ as described in Section 3.2. The networks are implemented using PyTorch [36]. We also use PyTorch in order to calculate the gradient $\nabla_{x}R^{\left(m\right)}$. We take $N_{\mathrm{iter}}=15$, s=0.25 and $x_{0}=\Phi^{\left(m\right)}A^{⊺}y$ in Algorithm 1 and compute the inverse ${\left(A^{⊺}A-s\mathrm{Id}\right)}^{-1}$ only once and then use it for all examples. We set α=0.015 for the noise-free case, α=0.016 for the low noise case and α=0.02 for the high noise cases, respectively, and selected a fixed β=15. We expect that the NETT functional will yield better results due to data consistency, which is mainly helpful outside the masked center diagonal.

**Figure 2:**
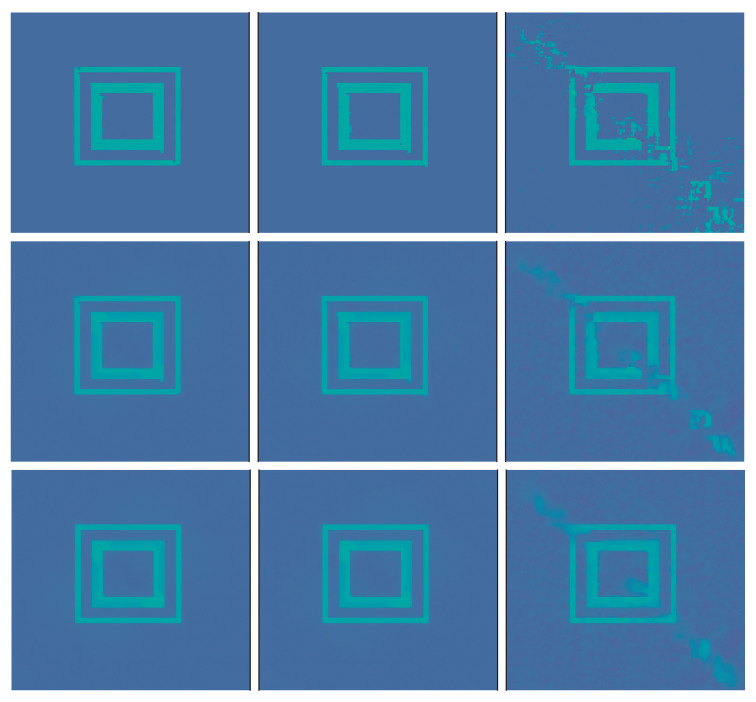
Top row: reconstructions using post-processing network $\Phi^{\left(1\right)}$. Middle row: NETT reconstructions using $R^{\left(1\right)}$. Bottom row: NETT reconstructions using $R^{\left(3\right)}$. From Left to Right: Reconstructions from data without noise, low noise (σ=0.01) and high noise (σ=0.1).

**Figure 3:**
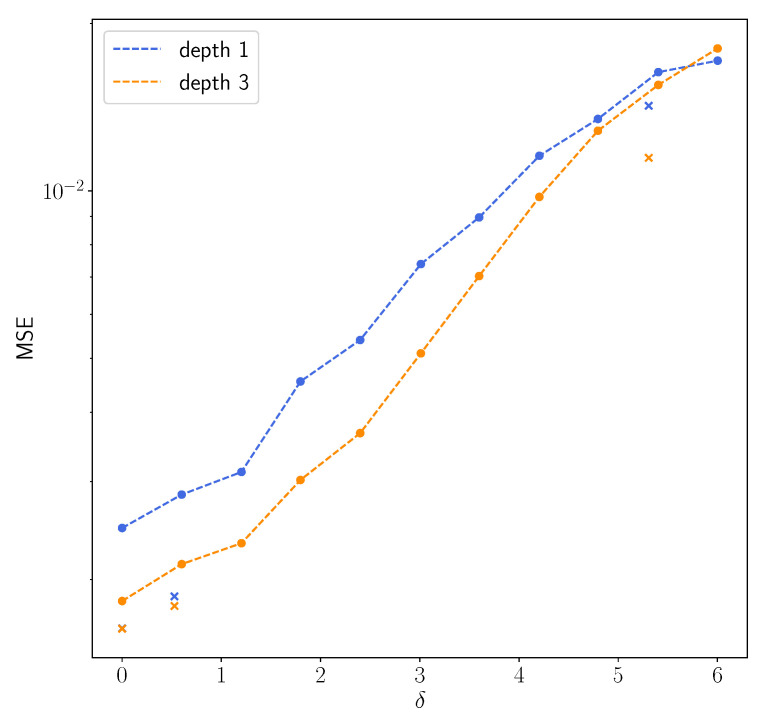
Semilogarithmic plot of the mean squared errors of the NETT using $R^{\left(1\right)}$ and $R^{\left(3\right)}$ depending on the noise level. The crosses are the values for the phantoms in Figure 2.

**Figure 4:**
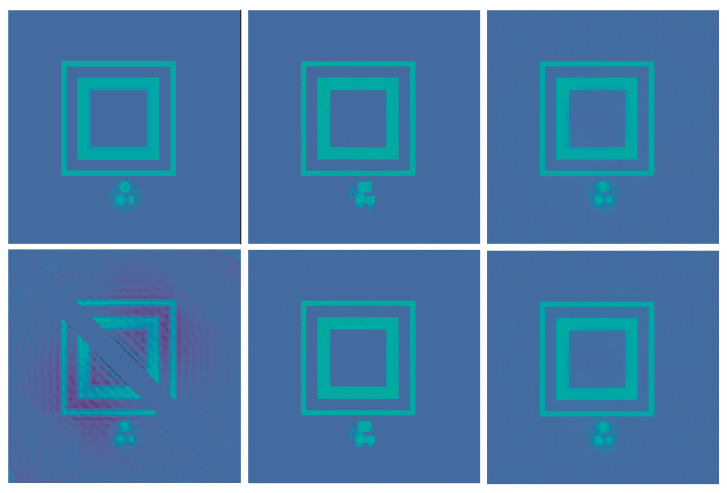
Left column: phantom with a structure not contained in the training data (**top**) and pseudo inverse reconstruction (**bottom**). Middle column: Post-processing reconstructions with $\Phi^{\left(3\right)}$ using exact (**top**) and noisy data (**bottom**). Right column: NETT reconstructions with $R^{\left(3\right)}$ using exact (**top**) and noisy data (**bottom**).

First we use the phantom from the testdata shown in Figure 1. The results using post processing and NETT are shown in Figure 2. One sees that all results with higher noise than used during training are not very good. This indicates that one should use similar noise as in the later applications even for the NETT. Figure 3 shows the average error using 10 test phantoms similar to the on in Figure 1. Careful numerical comparison of the numerical convergence rates and the theoretical results of Theorem 1 is an interesting aspect of further research. To investigate the stability of our method with respect to phantoms that are different from the training data we create a phantom with different structures as seen in Figure 4. As expected, the post processing network $\Phi^{\left(3\right)}$ is not really able to reconstruct the circles object, since it is quite different from the training data, but it also does not break down completely. On the other hand, the NETT approach yields good results due to data consistency.

## 4. Conclusions

We have analyzed the convergence a discretized NETT approach and derived the convergence rates under certain assumptions on the approximation quality of the involved operators. We performed numerical experiments using a limited data problem for PAT that is the combination of an inverse problem for the wave equation and an inpainting problem. To the best of our knowledge this is the first such problem studied with deep learning. The NETT approach yields better results that post processing for phantoms different from the training data. NETT still fails to recover some missing parts of the phantom in cases the data contains more noise than the training data. This highlights the relevance of using different regularizers for different noise levels. Finding ways to make the regularizers less dependent on the noise level used during training is a possible future research direction. Another interesting question is if this results can be combined with with approximation error estimates for neural networks e.g., [37,38]. It seems not obvious how these two approaches can be combined. Furthermore, studying how one can define neural network based regularizers that fulfill (12) might also be an interesting line of future research.

## Data Availability

Data and code are freely available upon request.
